# Analysis of the differential gene and protein expression profile of the rolled leaf mutant of transgenic rice (*Oryza sativa* L.)

**DOI:** 10.1371/journal.pone.0181378

**Published:** 2017-07-19

**Authors:** Qiuqiang Zhu, Shuguang Yu, Guanshui Chen, Lanlan Ke, Daren Pan

**Affiliations:** 1 Department of Life Science, Fujian Agriculture and Forest University, Fuzhou, China; 2 College of Chemistry and Pharmaceutical Sciences, Qingdao Agricultural University, Qingdao, China; The Institute of Genetics and Developmental Biology (IGDB) of the Chinese Academy of Sciences (CAS), China, CHINA

## Abstract

The importance of leaf rolling in rice (*Oryza sativa* L.) has been widely recognized. Although several studies have investigated rice leaf rolling and identified some related genes, knowledge of the molecular mechanism underlying rice leaf rolling, especially outward leaf rolling, is limited. Therefore, in this study, differential proteomics and gene expression profiling were used to analyze rolled leaf mutant of transgenic rice in order to investigate differentially expressed genes and proteins related to rice leaf rolling. To this end, 28 differentially expressed proteins related to rolling leaf traits were isolated and identified. Digital expression profiling detected 10 genes related to rice leaf rolling. Some of the proteins and genes detected are involved in lipid metabolism, which is related to the development of bulliform cells, such as phosphoinositide phospholipase C, *Mgll* gene, and *At4g26790* gene. The “omics”-level techniques were useful for simultaneously isolating several proteins and genes related to rice leaf rolling. In addition, the results of the analysis of differentially expressed proteins and genes were closely consistent with those from a corresponding functional analysis of cellular mechanisms; our study findings might form the basis for further research on the molecular mechanisms underlying rice leaf rolling.

## Introduction

Rice (*Oryza sativa* L.) is one of the most important grain crops worldwide, particularly in populous countries such as China. Under conditions of limited farmland, stabilizing and improving rice yield per unit area is the most effective way to improve overall rice yields. Moreover, improving the quality of rice plant is important for increasing the unit yield of rice. The erectness of rice leaves and their proper rolling are important factors that determine ideal type of rice plants [1,2]. Proper inward rolling of the leaves enables them to be upright and not drooping, thereby reducing mutual shielding between the leaf blades and improving the light transmittance (transmission efficiency) of the community [3]. In recent years, several studies have been focusing on the rice leaf rolling trait, specifically the isolation of genes related to leaf rolling and the investigation of the mechanisms underlying rice leaf rolling.

To date, 13 genes associated with rice leaf rolling have been isolated or cloned [4–16]. The cytological mechanism of leaf rolling has been found to be largely related to the abnormal development of bulliform cells. The genes *NRL1* and *RL14* encode cellulose synthase and 2GO-Fe (II) dioxygenase, respectively [6], and play a positive role in the regulation of bulliform cell development. In mutant rice plants that lack these two genes, shrinkage is noted in the area of the bulliform cells, thereby causing the inward rolling of rice leaves. In addition, the gene *ACL1* encodes an unknown protein with a conserved functional domain; the gene *OsZHD1* encodes a domain transcription factor with homologous zinc finger structure. These genes also play a positive role in the regulation of bulliform cell development, and over-expression of these two genes results in an increased number of bulliform cells, thereby causing outward rolling of rice leaves [7,16]. The gene *SLL1* encodes a SHAQKYF-class transcription factor belonging to the MYB family. Owing to developmental defects in the parietal cells on the adaxial side and the abnormal development of bulliform cells on the abaxial side, the sll1 mutant shows inward rolling of the rice blade [11]. The gene *Roc5* negatively regulates the formation and development of bulliform cells, and inhibition of its expression leads to an increase in the number of upper epidermal bulliform cells, thereby causing outward rolling of rice leaves [13]. The gene *SRL1* encodes a putative glycosyl phosphatidylinositol-anchored protein. This gene negatively regulates bulliform cell development in rice. Inhibition of its expression leads to an increased number of bulliform cells [15]. Thus, bulliform cell development has an important effect on the maintenance of rice leaf morphology.

Investigating the molecular mechanism underlying bulliform cell development is crucial for understanding that underlying rice leaf rolling. However, the cloned genes that are related to rice leaf rolling failed to yield an effective molecular network to comprehensively explain the molecular mechanism of bulliform cell development. Therefore, for a holistic understanding of this mechanism, more relevant genes should be isolated so that a complete molecular network can be obtained.

Therefore, in the present study, stable high-generation mutant transgenic rice plants with outward rolling leaf (Rolled) were used as the experimental group, whereas cultivated rice variety MH 86 (WT) and transgenic plants with unrolled leaf (Unrolled) were used as the control groups; differential proteomic analysis based on two-dimensional gel electrophoresis and digital gene expression profiling analysis based on high-throughput sequencing were used in order to analyze the differentially expressed proteins and genes related to rice rolled leaf mutant. The information on proteins and genes related to rice leaf rolling identified in the present study can be closely linked to the corresponding cytological mechanism, thereby forming a basis for further studies on the molecular mechanism underlying rice leaf rolling.

## Materials and methods

### Plant materials and growth condition

Plants used in this study were stable high-generation mutants of *3α-HSD* transgenic rice with outward rolled leaf (Rolled), stable high-generation mutants of *3α-HSD* transgenic rice with unrolled leaf (Unrolled), and MH 86 plants of transgenic receptor rice variety (WT) that are preserved in our laboratory. The exogenous *3α-HSD* gene is *3α*-hydroxysteroid dehydrogenase/carbonyl reductase gene, which encodes the most important enzyme in the bacterium *Comamonas testosteroni*. The product of exogenous gene can effectively degrade steroid compounds in environmental contaminants. The rolled leaf mutant begins to show leaf rolling trait from the fourth leaf. The leaf shape is normal in the seedling period. Experimental plants were grown in isolation in the experimental farm fields of Fujian Agriculture and Forestry University. They are under field management according to conventional methods.

### Research background of testing materials

Previous studies in our laboratory have shown that the chlorophyll content and photosynthetic efficiency, and plant height, ear length, and other agronomic trait indices of rolled leaf mutant are lower than those of normal-leaf plants (WT and Unrolled). The cytological mechanism underlying the leaf rolling trait is the increase in the number of bulliform cells in the upper epidermis. The rolled leaf trait of the rolled leaf mutant is because of the exogenous T-DNA insertion. This exogenous T-DNA has an inhibitory effect on the expression of genes near the insertion site. The insertion site of the exogenous T-DNA is at 7.5M on the long arm of chromosome 9 of rice, which is in a non-gene region.

### Two-dimensional electrophoresis of proteins

When rolled leaves had not yet appeared in seedlings, the second leaf of each of the three plants (WT, Unrolled, and Rolled) was collected. Next, at the interim of the seedling stage (rolling), samples were collected when the rolled leaf trait was just observed. The fourth or fifth leaf of each of the three plants was collected. For each material, leaves from six plants were collected to form mixed samples, which were stored at ultra-low temperature for the subsequent two-dimensional electrophoresis experiment.

The trichloroacetic acid-acetone precipitation method was used for the extraction of total protein from rice leaves. Homemade immobilized pH gradient (IPG) strip adhesive was used for isoelectric focusing, followed by SDS-PAGE and staining by using the silver staining method. The gels were scanned using ScanMaker 5. PDQuest 8.0 software (Bio-Rad) was employed to analyze the images. Three well-separated gels of each sample were used to create “replicate groups.” After statistic, quantitative, and qualitative analyses were completed, the spots from the Boolean sets were compared among three biological replicates. Only spots showing reproducible change patterns were considered to be differentially expressed proteins.

In this study, because the rice rolled leaf mutant did not show rolled leaf trait at the seedling stage or trefoil stage, but began to show this trait at the fourth leaf stage, and the rolled leaf mutant was a transgenic mutant, we excluded the influence of differential protein expression caused by exogenous *3α-HSD* expression. Therefore, in order to identify differentially expressed proteins relevant to the rolled leaf, two-dimensional electrophoresis profiles of rice leaves in seedling and rolling periods were analyzed together. Only the protein spots that were differentially expressed at the rolling stage, but not at the seedling stage, were selected. The protein spots that were differentially expressed not only between WT and Rolled and between Rolled and Unrolled, but also between WT and Unrolled, were removed, because these proteins might be induced by exogenous gene expression, and hence could be unrelated to the rolled leaf trait.

The soluble total protein extracted from rice leaf was first subjected to protein spot isolation by using two-dimensional polyacrylamide gel electrophoresis. The differential protein spots obtained from precipitation were subjected to in-gel enzymatic degradation in situ. Subsequently, mass spectrometry was used for the peptide mass fingerprinting (PMF) of protein spots. Database searching and both peptide mass fingerprinting (PMF) and tandem mass spectrometry (MS/MS) were performed using MASCOT (version 2.3) program included in GPS Explorer software (version 3.6). The database was set to the National Center for Biotechnology non-redundant (NCBInr; updated on May 5, 2015), which contained 1,850,050 sequences and 642,453,415 residues; species restriction to Viridiplantae (green plants); at most two missed cleavage sites; MS and MS/MS tolerance were set as 100 ppm and 0.6 Da, respectively; PMF of protein spots and the theoretical PMF of protein or nucleic acid sequence in the protein database were compared and analyzed to identify proteins. After database search, protein spots with a score greater than 65 points were the spots of successful identification (*P* < 0.05).

The identified differentially expressed proteins were classified by function according to Gene Ontology (GO), the international standard gene function classification system. The GO project provides an ontology of defined terms representing gene product properties, which allows effective annotation of gene products. The ontology covers three domains: cellular component, molecular function, and biological process. The GO ontology file is freely available from the GO website in many formats.

### Digital expression profiling analysis

Because the leaves of the rolled leaf mutant show an outward rolling trait after unfolding, newly folded core leaves growing at the tillering stage were collected for RNA extraction. The leaves of each of WT, Rolled, and Unrolled plants were collected as samples for RNA extraction. First, agarose gel electrophoresis was used to assess the degradation of the extracted total RNA and the contamination. Subsequently, ultraviolet spectrophotometry was used to analyze the purity of the extracted total RNA. Qubit was used for precise quantitative analysis of the concentration of the extracted total RNA, based on the analysis of the ratio of absorbance at 260 nm to that at 280 nm (OD_260_/OD_280_). Finally, Agilent 2100 was used to accurately determine the integrity of total RNA. The total RNA deemed to be of high quality was used for high-throughput sequencing analysis. A sequencing platform based on Illumina HiSeq2000 technology was used to generate a conventional transcriptome library for SE100 (single-end sequencing for 100 bp) single-terminal sequencing to obtain digital gene expression (DGE) sequencing data.

Reads Per Kilo bases per Million reads (RPKM) is the measurement of the relative gene expression level. TopHat2 was used to complete the sequence alignment, and the mismatch was set to 2. The diagrammatic sketch of TopHat2 is shown in S1 Fig. The reference genome sequence was the rice genome sequence. TMM was used to standardize read count data, and then DEGseq was used to complete the variance analysis.

For samples of no biological duplication, biological variations were eliminated using two levels of both fold change and significant level to evaluate and screen differentially expressed genes. Threshold value was set to |log_2_(FoldChange)| > 1 and q value as <0.005.

## Results

### Analysis and identification of two-dimensional electrophoresis profile of differentially expressed proteins

Analysis of the obtained two-dimensional electrophoresis profiles suggested that more than 700 protein spots were present on each profile. A total of 181 differentially expressed protein spots were identified according to the final observation after analysis and comparison.

**Fig 1:**
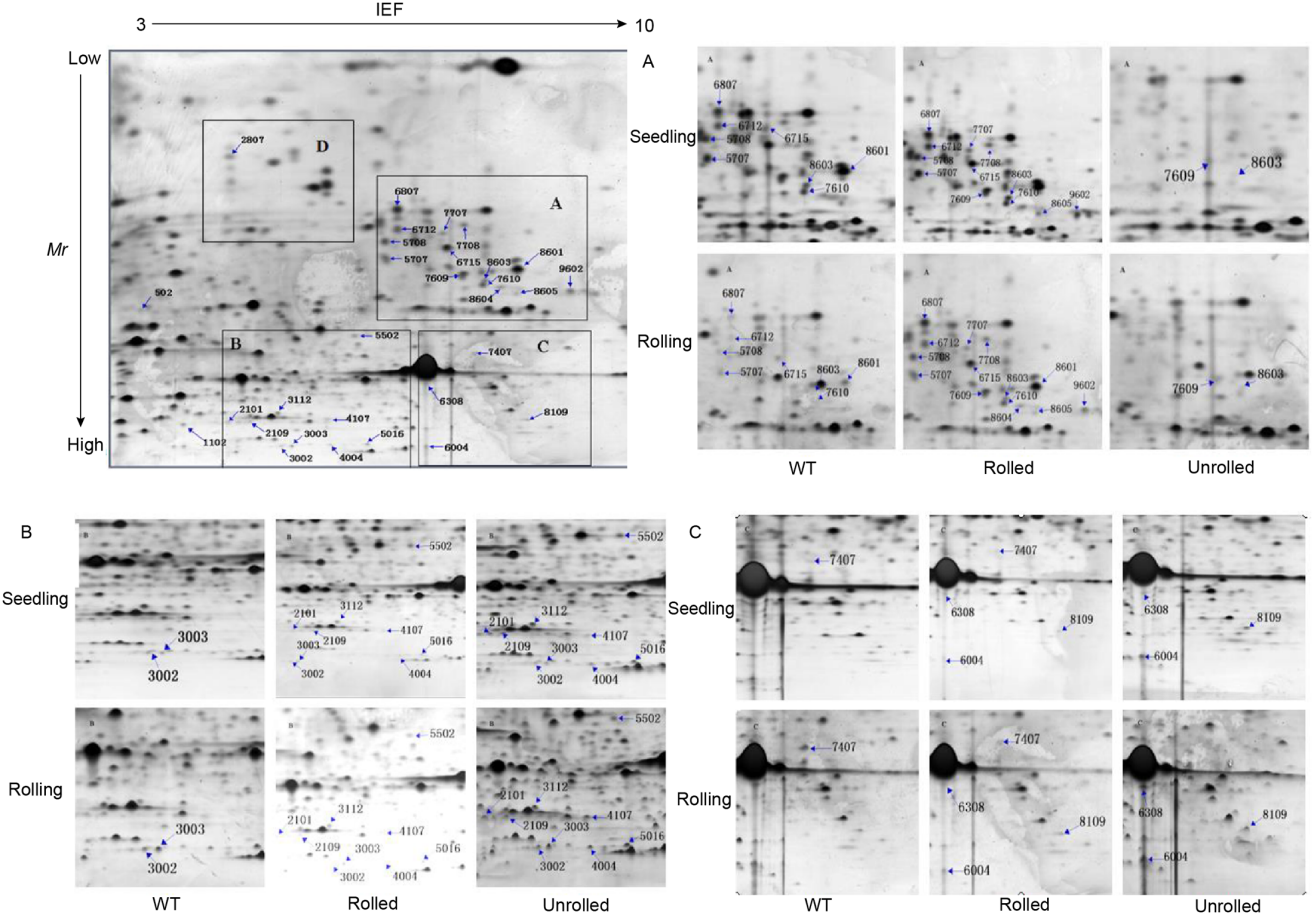
Electrophoretograms of rice leaf proteins identified using two-dimensional electrophoresis. The four boxes A, B, C, and D in the panel (upper-left comer) represent four areas where differentially expressed proteins are intensive. The small figures in the other three panels A, B, and C represents the corresponding areas (A, B, and C) of the first panel (upper-left corner); the numbers indicate the number of differentially expressed proteins. WT, Rolled, and Unrolled represent different experimental materials of rice plants. Seedling and Rolling represent two periods.

Eventually, after screening, 34 differentially expressed protein spots that were not related to the rolled leaf trait were obtained (S1 Table and Fig 1). There were 17 down-regulated and 10 up-regulated proteins in the rolled leaf mutant (Rolled). Five proteins were expressed only in rolled leaf mutant (Rolled), whereas 2 proteins were expressed only in MH 86 cultivated rice (WT).

The 34 protein spots obtained from differential expression analysis of proteins were analyzed and identified by mass spectrometry. Finally, 28 differentially expressed protein spots were successfully identified. The specific results are shown in S2 Table.

### Functional classification of differentially expressed proteins

The detailed classification of differentially expressed proteins is shown in S3 Table. The majority of differential proteins were those related to energy metabolism. The number of different isoforms of the ribulose bisphosphate carboxylase large subunit was as high as 15. In addition, photosynthesis-related transketolase 1 and chloroplast protein pyruvate orthophosphate dikinase, carbohydrate metabolism-associated glucuronidase, and 2 ATP synthase subunits were located in the chloroplast. A total of 23 proteins were associated with energy metabolism. In addition, there were protein folding-associated cis-trans peptidyl-proline isomerase proteins, diphosphate synthases that are essential for chloroplast development, glyoxylate cycle-associated aconitate synthase, aconitate synthase associated with isocitrate metabolism, amino acid metabolism-associated aspartate aminotransferase, protein synthesis-associated elongation factor, protein transport-associated chloroplast envelope protein, and ester-catabolism-associated phosphoinositide phospholipase C.

Chlorophyll content and photosynthetic efficiency of the rolled leaf mutant were lower than those of the normal-leaf plant. Correspondingly, 4 proteins with down-regulated expression were found: chloroplast development-related protein diphosphate synthase, protein transport-associated chloroplast envelope protein, photosynthesis-related transketolase 1, and chloroplast protein pyruvate phosphate dikinase. The plant height, ear length, and other agronomic indices of rolled leaf mutant were lower than those of normal leaf plants. Correspondingly, 7 energy-related proteins were down-regulated or not expressed. The remaining 4 proteins with down-regulated expression, 9 proteins with up-regulated expression, and 4 proteins that were expressed only in the Rolled mutant might be associated with the rolled leaf trait. Of these, the phosphoinositide phospholipase C is involved in lipid metabolism. Bulliform cells are highly vacuolated parenchyma cells. Their tonoplasts are larger than those of ordinary cells in terms of area; phospholipids are essential components of the tonoplast, accounting for more than 50% of the vacuolar membrane lipids. Thus, presumably, phosphoinositide phospholipase C might affect bulliform cell development by controlling tonoplast development. The protein folding-related peptide group-proline cis-trans isomerase might be involved in the regulation of the biosynthesis of the cell wall structural protein called the proline-rich protein. Bulliform cells are thin-walled cells, indicating that they contain only the primary cell wall, but no secondary cell wall. Ordinary leaf epidermal cells have secondary walls. Therefore, peptidyl-proline cis-trans isomerase might affect bulliform cell development by controlling the development of secondary cell wall. Phosphoinositide phospholipase C is expressed only in the rolled leaf mutant, and proline peptidyl cis-trans isomerase is downregulated. Phosphoinositide phospholipase C and proline peptidyl cis-trans isomerase cause outward rolling of rice leaves by influencing bulliform cell development in the rice leaf mutant. Thus, bulliform cell development is positively regulated by phosphoinositide phospholipase C, but negatively by proline peptidyl cis-trans isomerase.

### Analysis of high-throughput sequencing data

The overall quality of sequencing data is shown in S4 Table. Error rate was only 0.06%; the error rate is very low, and sequencing quality Q30 was greater than 85%, higher than the official sequencing quality indicators of Illumina (Q30 ≥ 80%). This implies that sequencing quality is good, and they can be used for subsequent analysis.

The result of sequence alignment by TopHat2 is shown in S5 Table; the percentage of total mapped reads or fragments was higher than 70%, suggesting that the reference genome is suitable. For samples of no biological duplication, TMM was used to standardize read count data, and then DEGseq was used to complete the variance analysis. The statistical analysis of differentially expressed genes is shown in S6 Table.

### Venn diagram of differentially expressed genes

**Fig 2:**
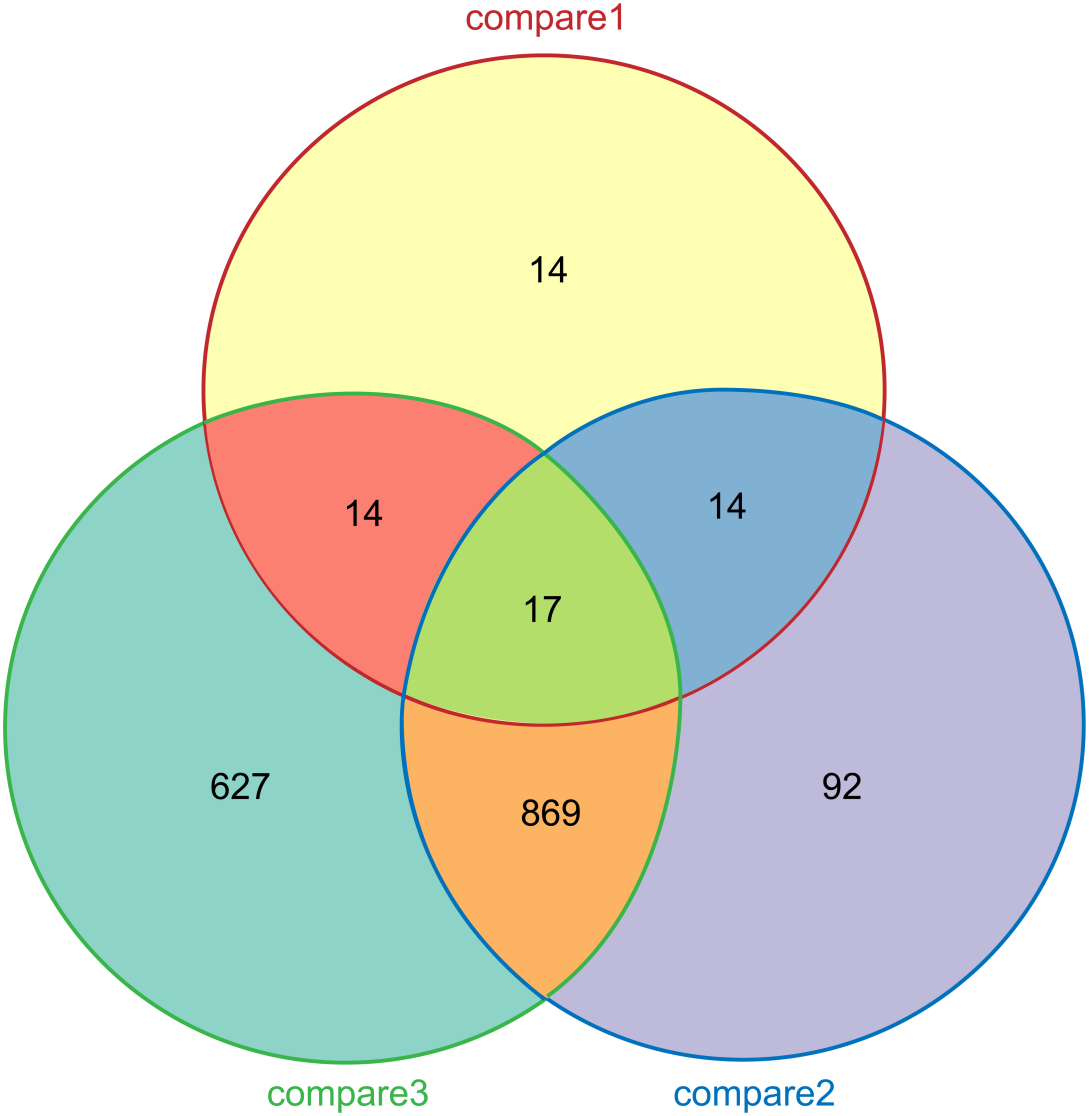
Venn diagram of differentially expressed genes. WT, Unrolled, and Rolled are different experimental samples of rice plant. Compare 1 represents differentially expressed genes between Rolled and WT; compare 2, between Unrolled and WT; and compare 3, between Unrolled and Rolled. The figures denote the numbers of differentially expressed genes.

The Venn diagram shows the number of differentially expressed genes between every two samples and the number of differentially expressed genes common to samples. The number of genes in the intersecting area of compares 1 and 3 (with differential expression between WT and Rolled and between Rolled and Unrolled) is 31 (Fig 2). Other than the 17 genes with differential expression between WT and Unrolled (these differences might be attributed to the expression of exogenous genes), the remaining 14 genes have the largest correlation with the rolled leaf mutant of rice. Therefore, the 14 genes in the intersecting area between compares 1 and 3 were analyzed. Database retrieval did not reveal any relevant information for 4 of them. Finally, 10 differentially expressed genes that might be related to the rolled leaf trait were identified. The detailed results are shown in Table 1.

**Table 1:** Differentially expressed genes.

Gene no.	Gene name	Protein name	Function	Organism	Relative expression		
					Rolled	WT	Unrolled
BGIOSGA004822 (↑)	*CYP86A1*	Cytochrome P450 86A1	Suberin/casparian strip/suberinite biosynthesis	*Arabidopsis thaliana*	49.105	12.822	12.576
BGIOSGA011125 (↓)	*PRP2*	Proline-rich protein 2	Plant cell wall tissue	*Arabidopsis thaliana*	54.207	121.864	122.828
BGIOSGA006323 (↓)	*Mgll*	Monoglyceride lipase	Regulate fatty acid concentration	*Rattus norvegicus (Rat)*	2.870	41.406	27.467
BGIOSGA011503 (↓)	*GSTF10*	Glutathione S-transferase F10	Cell wall proteins; chloroplast proteins	*Arabidopsis thaliana*	34.278	91.281	131.771
BGIOSGA011504 (↓)	*GSTF10*	Glutathione S-transferase F10	Cell wall proteins; chloroplast proteins	*Arabidopsis thaliana*	34.278	91.281	131.771
BGIOSGA021464 (↑)	*PER11*	Peroxidase 11	Lignin synthesis and degradation; suberification	*Arabidopsis thaliana*	44.960	13.527	16.349
BGIOSGA013555 (↓)	*Not Applicable*	Short-chain type dehydrogenase	Oxidoreductase	*Picea abies (Norway spruce)*	34.597	100.221	129.815
BGIOSGA020820 (↑)	*At4g26790*	GDSL esterase /lipase At4g26790		*Arabidopsis thaliana*	151.143	56.109	38.786
BGIOSGA028855 (↑)	*BHLH14*	Transcription factor bHLH14		*Arabidopsis thaliana*	151.143	39.641	60.086
BGIOSGA028962 (↓)	*At1g66830*	Probable inactive leucine-rich repeat receptor-like protein kinase At1g66830		*Arabidopsis thaliana*	73.977	152.919	220.084

Note:

^(↑)^ represents up-regulation in rolled leaf mutants (Rolled),

^(↓)^ represents down-regulation in rolled leaf mutants. RPKM (reads per kilo bases per million reads) is the measurement of relative expression.

In all, 6 differentially expressed genes showed down-regulated expression in the rolled leaf mutant and 4 showed up-regulated expression (Table 1). Among the genes with down-regulated expression, those related to energy metabolism and chlorophyll and photosynthesis might be correlated with the relatively low chlorophyll content and photosynthetic efficiency and agronomic trait indices of the mutant. For example, the gene *GSTF10* encodes a chloroplast protein. Therefore, the other genes showing down-regulated and up-regulated expression might be correlated with the rolled leaf trait and could be associated with bulliform cell development in rice leaf.

Cytochrome P450 86A1 encoded by the *CYP86A1* gene and peroxidase 11 encoded by the *PER11* gene are involved in the biosynthesis of suberin and lignin, which are the main components of the secondary cell wall of plants and serve to increase cell wall strength. Bulliform cells are thin-walled cells, with only the primary cell wall. Therefore, cytochrome P450 86A1 might play a role in bulliform cell development by participating in the morphogenesis of the secondary cell wall. Because the genes *CYP86A1* and *PER11* both showed up-regulated expression in the rolled leaf mutant, they might play a positive regulatory role in bulliform cell development of rice leaves. Plant cell walls contain several proteins. The proteins that contribute to the cell wall structure have highly repetitive amino acid sequences and are highly glycosylated. The gene *PRP2* encoding proline-rich protein 2 and the gene *At1g66830* gene encoding leucine-rich repetitive receptor-like protein kinase might be involved in cell wall morphogenesis in rice leaf, thus playing a role in bulliform cell development. The gene *GSTF10* encoding glutathione transferase F10 is involved in the synthesis of cell wall proteins and might also play a similar role. Because *PRP2*, *At1g66830*, and *GSTF10* show down-regulated expression in the rolled leaf mutant, they might positively regulate bulliform cell wall morphogenesis in rice. The highly vacuolated bulliform cells contain phospholipids that are an important component of the tonoplast. Phospholipids are mainly divided into two types, glycerol and sphingomyelin. Our results suggested that *Mgll* genes, encoding monoglyceride lipase, and *At4g26790*, encoding GDSL lipase, might be involved in tonoplast morphogenesis, thereby potentially playing a role in bulliform cell development. Because *Mgll* showed down-regulated expression in the rolled leaf mutant, it probably negatively regulates tonoplast morphogenesis in bulliform cells of rice. In contrast, *At4g26790* shows up-regulated expression in the rolled leaf mutant, and thus might positively regulate tonoplast morphogenesis. The function of *BHLH14* encoding the transcription factor bHLH14 is not known; it shows up-regulated expression in the rolled leaf mutant and might also be associated with the rolled leaf trait.

## Discussion

In this study, stable high-generation mutant transgenic rice plants with the outward rolling leaf trait (Rolled) were used as the study material; normal-leaf plants of rice cultivar MH 86 (WT) and transgenic rice with normal leaves (Unrolled) were used as the control group. The differential proteomic analysis of the three materials revealed 13 proteins related to the rolled leaf trait of rice. Analysis of the differential expression profile of the three materials revealed 10 genes related to the rolled leaf trait of rice. Of these, *CYP86A1* and *PER11* positively regulated bulliform cell development. *PRP2*, *At1g66830*, and *GSTF10* positively regulated cell wall morphogenesis in bulliform cells. *Mgll* and *At4g26790* negatively and positively regulated, respectively, the morphogenesis of tonoplasts in bulliform cells. The differentially expressed genes and proteins were involved in lipid metabolism, which is consistent with the findings of histological analysis that outward curling is caused by the abnormal development of bulliform cells.

Our results provide novel evidence of the molecular mechanism underlying rice leaf rolling, especially the molecular mechanism of bulliform cell development in rice. To date, very few studies have investigated the molecular mechanism of rice bulliform cell development. The genes *NRL1* and *RL14* positively regulated bulliform cell development. The mutant rice plant lacking these two genes showed shrinkage in the area bulliform cells, and hence inward rolling of leaves [6]. The genes *ACL1* and *OsZHD1* also positively regulated rice bulliform cell development. Over-expression of these two genes leads to an increase in the number of bulliform cells in rice, thereby causing outward rolling of leaves [7,16]. The gene *Roc5* negatively regulated bulliform cell formation and development. Lack of this gene leads to the increase in the number of upper epidermal bulliform cells, thereby causing outward rolling of rice leaves [13]. The gene *SRL1* expresses a putative glycosyl phosphatidylinositol-anchored protein. This gene plays a negative role in the regulation of rice bulliform cell development. A lack of this gene leads to an increase in the number of bulliform cells [15].

Technically, most previous studies on the molecular mechanisms underlying rice leaf rolling identified relevant genes by map-based cloning, which can provide data on only one gene at a time. In contrast, the present study used proteomics techniques, in order to identify multiple genes simultaneously. These techniques can be used to more comprehensively analyze genes related to rice leaf rolling. According to our results, the identified genes related to bulliform cell development in rice leaf were different from those identified in previous studies. This is mainly because the site of mutation used in the present study was different from those used in other studies. Therefore, the resultant gene expression was not the same. In addition, the genes identified in the present study are primarily associated with tonoplast and cell wall development in bulliform cells. Therefore, the results of the present study could better link the cytological and molecular mechanisms of bulliform cell development. In our study, multiple genes associated with rice rolled leaf were newly identified simultaneously. This fact further illustrates the complexity of the molecular regulatory network of bulliform cells in rice leaves. Further studies are warranted to explore the molecular regulatory mechanism of bulliform cells and to devise strategies for improving the quality of rice plants.

## Supporting information

S1 Fig Diagrammatic sketch of TopHat2. (DOC)

S1 Table Statistical analysis of the differentially expressed proteins in rice leaf. (DOCX)

S2 Table Differentially expressed proteins identified by tandem mass spectrometry. (DOCX)

S3 Table Gene Ontology classification of the differentially expressed proteins. (DOCX)

S4 Table Summary of the data obtained from high-throughput sequencing. (DOCX)

S5 Table Sequence alignment of reads to the reference genome. (DOCX)

S6 Table Statistical analysis of differentially expressed genes. (DOCX)
