# Supplementary figures and images for: Analysis of the differential gene and protein expression profile of the rolled leaf mutant of transgenic rice (Oryza sativa L.)
Source: PLoS One. 2017 Jul 19;12(7):e0181378. doi: 10.1371/journal.pone.0181378 (PMC5517006; doi:10.1371/journal.pone.0181378)

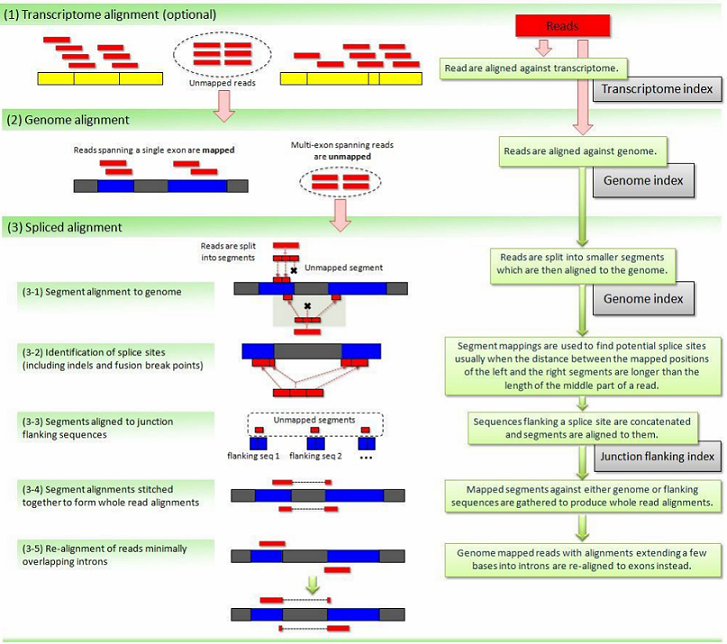


**S1 Figure.** **Diagrammatic sketch of TopHat2.**

Supplement: S1 Fig — (DOC) [file pone.0181378.s001.doc]
