# Supplementary material for: Analysis of the differential gene and protein expression profile of the rolled leaf mutant of transgenic rice (Oryza sativa L.)
Source: PLoS One. 2017 Jul 19;12(7):e0181378. doi: 10.1371/journal.pone.0181378 (PMC5517006; doi:10.1371/journal.pone.0181378)
Supplement: S1 Table — (DOCX) [file pone.0181378.s002.docx]

**S1** **Table. Statistical analysis of the differentially expressed proteins in rice leaf.**

| **Differential expression of proteins** | | **Spot no.** |
| --- | --- | --- |
| Differentially expressed proteins only detected in the rolling period, but not in the seedling period | Expression was lower in WT-rolling than in Rolled-rolling | 5707, 5708, 6712, 6715, 6807, 7610, 8601, 8603 |
|  | Expression was higher in WT-rolling than in Rolled-rolling | 2807, 3002, 3003, 7407 |
|  | Only detected in WT-rolling | None |
|  | Only detected in Rolled-rolling | 7707, 7708, 8605, 8604, 9602 |
|  | Expression was lower in Unrolled-rolling than in Rolled-rolling | 7609, 8603 |
|  | Expression was higher in Unrolled-rolling than in Rolled-rolling | 502, 1102, 2101, 2109, 3002, 3003, 3112, 4004, 4107, 5016, 5502, 6004, 6308, 8109, |
|  | Only detected in Unrolled-rolling | None |
|  | Only detected in Rolled-rolling | None |
| Differentially expressed proteins only detected in WT vs. Rolled, but not in WT vs. Unrolled | Expression was lower in WT-rolled than in Rolled-rolled | 3112 |
|  | Expression was higher in WT-rolled than in Rolled-rolled | 7611, 2807 |
|  | Only detected in WT-rolled | 5807, 6604 |
|  | Only detected in Rolled-rolled | None |
|  | Expression was lower in Unrolled-rolled than in Rolled-rolled | None |
|  | Expression was higher in Unrolled-rolled than in Rolled-rolled | 7611, 2807 |
|  | Only detected in Unrolled-rolled | None |
|  | Only detected in Rolled-rolled | None |

Note: Rolled, Unrolled, and WT represent different treatments; rolling and rolled represent different growth stages.
