# Supplementary material for: Analysis of the differential gene and protein expression profile of the rolled leaf mutant of transgenic rice (Oryza sativa L.)
Source: PLoS One. 2017 Jul 19;12(7):e0181378. doi: 10.1371/journal.pone.0181378 (PMC5517006; doi:10.1371/journal.pone.0181378)
Supplement: S2 Table — (DOCX) [file pone.0181378.s003.docx]

**S2 Table.** **Differentially expressed proteins identified by tandem mass spectrometry.**

| **Spot no.** | **NCBI**  **accession no.** | **Protein name** | **Organism** | **Mr** | **pI** | **Score** |
| --- | --- | --- | --- | --- | --- | --- |
| 0502 | gi\|50508306 | Putative peptidyl-prolyl cis-trans isomerase protein | *Oryza sativa* subsp. *japonica* | 46062 | 5.31 | 82 |
| 2109 | gi\|75261413 | 4-hydroxy-3-methylbut-2-en-1-yl diphosphate synthase, chloroplastic | *Oryza sativa* subsp. *japonica* | 82109 | 5.64 | 207 |
| 2807 | gi\|290585768 | Ribulose bisphosphate carboxylase large chain | *Oryza sativa* | 26211 | 7.00 | 241 |
| 3003 | gi\|75254569 | Pyruvate, phosphate dikinase 1, chloroplastic | *Oryza sativa* subsp. *japonica* | 102722 | 5.98 | 70 |
| 3112 | gi\|55297190 | Putative transketolase 1 | *Oryza sativa* subsp. *japonica* | 68836 | 5.43 | 147 |
| 4004 | gi\|75225211 | Putative aconitate hydratase, cytoplasmic | *Oryza sativa* subsp. *japonica* | 98021 | 5.67 | 76 |
| 4107 | gi\|14594803 | Beta-glucuronidase | *Arabidopsis thaliana* | 68390 | 5.24 | 179 |
| 5016 | gi\|108706066 | Aconitate hydratase, cytoplasmic, putative | *Oryza sativa* subsp. japonica | 106235 | 6.45 | 186 |
| 5502 | gi\|29468084 | Aspartate aminotransferase | *Oryza sativa* | 45845 | 5.90 | 117 |
| 5707 | gi\|109156602 | Ribulose bisphosphate carboxylase large chain | *Oryza sativa* subsp. *indica* | 53667 | 6.33 | 216 |
| 5708 | gi\|110288945 | Ribulose bisphosphate carboxylase large chain, putative | *Oryza sativa* subsp. *japonica* | 40249 | 8.51 | 226 |
| 5807 | gi\|476752 | Ribulose bisphosphate carboxylase large chain | *Oryza sativa* | 45102 | 8.43 | 122 |
| 6004 | gi\|119395218 | Elongation factor | *Oryza sativa* subsp. *japonica* | 93913 | 5.85 | 69 |
| 6308 | gi\|171259300 | ATP synthase CF1 alpha chain ,chloroplast | *Oryza sativa* subsp. *indica* | 55630 | 5.95 | 409 |
| 6604 | gi\|290585768 | Ribulose bisphosphate carboxylase large chain | *Oryza sativa* | 26211 | 6.33 | 76 |
| 6712 | gi\|109156602 | Ribulose bisphosphate carboxylase large chain | *Oryza sativa* | 53667 | 8.51 | 145 |
| 6715 | gi\|110288945 | Ribulose bisphosphate carboxylase large chain, putative | *Oryza sativa* | 40249 | 6.35 | 158 |
| 6807 | gi\|255673766 | Os01g0791033 protein | *Oryza sativa* subsp. *japonica* | 28824 | 9.04 | 135 |
| **Spot no.** | **NCBI**  **accession no.** | **Protein name** | **Organism** | **Mr** | **pI** | **Score** |
| 7407 | gi\|108862318 | Ribulose bisphosphate carboxylase large chain, putative | *Oryza sativa* subsp. *japonica* | 56034 | 7.00 | 96 |
| 7609 | gi\|290585768 | Ribulose bisphosphate carboxylase large chain | *Oryza sativa* | 26211 | 7.00 | 211 |
| 7610 | gi\|109156602 | Ribulose bisphosphate carboxylase large chain | *Oryza sativa* | 53667 | 6.33 | 102 |
| 7708 | gi\|11466794 | ATP synthase subunit beta, chloroplastic | *Oryza sativa* subsp. *japonica* | 53980 | 5.47 | 156 |
| 8109 | gi\|108707416 | Chloroplastic outer envelope membrane protein, putative | *Oryza sativa* subsp. *japonica* | 87632 | 8.50 | 327 |
| 8601 | gi\|290585768 | Ribulose bisphosphate carboxylase large chain | *Oryza sativa* | 26211 | 7.00 | 301 |
| 8603 | gi\|108862318 | Ribulose bisphosphate carboxylase large chain, putative | *Oryza sativa* subsp. *japonica* | 56034 | 9.04 | 188 |
| 8604 | gi\|388556101 | Ribulose bisphosphate carboxylase large chain | *Scenedesmus obliquus* | 16600 | 7.00 | 81 |
| 8605 | gi\|290585768 | Ribulose bisphosphate carboxylase large chain | *Oryza sativa* | 26211 | 7.00 | 141 |
| 9602 | gi\|355489828 | Phosphoinositide phospholipase C | *Medicago truncatula* | 64905 | 6.35 | 94 |

Note: Mr is the relative molecular weight.
