# Supplementary material for: Analysis of the differential gene and protein expression profile of the rolled leaf mutant of transgenic rice (Oryza sativa L.)
Source: PLoS One. 2017 Jul 19;12(7):e0181378. doi: 10.1371/journal.pone.0181378 (PMC5517006; doi:10.1371/journal.pone.0181378)
Supplement: S3 Table — (DOCX) [file pone.0181378.s004.docx]

**S3 Table.** **Gene Ontology classification of the differentially expressed proteins.**

| **Spot no.** | **Protein name** | **GO** | | |
| --- | --- | --- | --- | --- |
|  |  | **Molecular function** | **Biological progress** | **Cellular component** |
| 0502 (↓) | Putative peptidyl-prolyl cis-trans isomerase | peptidyl-prolyl cis-trans isomerase activity | protein folding |  |
| 2109 (↓) | 4-hydroxy-3-methylbut-2-en-1-yl diphosphate synthase, chloroplastic | 4-hydroxy-3-methylbut-2-en-1-yl diphosphate synthase activity | many biological progresses | chloroplast envelope and stroma |
| 2807 (↓) | Ribulose bisphosphate carboxylase large chain | magnesium ion binding; monooxygenase activity; ribulose-bisphosphate carboxylase activity | photorespiration; reductive pentose-phosphate cycle | chloroplast |
| 3003 (↓) | Pyruvate, phosphate dikinase 1, chloroplastic | ATP binding; kinase activity; metal ion binding; pyruvate; phosphate dikinase activity | photosynthesis | chloroplast stroma; cytosol; nucleus |
| 3112 (↓) | Putative transketolase 1 | transketolase activity |  |  |
| 4004 (↓) | Putative aconitate hydratase, cytoplasmic | 4 iron, 4 sulfur cluster binding; aconitate hydratase activity | glyoxylate cycle | cytoplasm |
| 4107 (↓) | Beta-glucuronidase | hydrolase activity, hydrolyzing *O*-glycosyl compounds | carbohydrate metabolic process |  |
| 5016 (↓) | Aconitate hydratase, cytoplasmic, putative | 4 iron, 4 sulfur cluster binding; aconitate hydratase activity; copper ion binding; isocitrate hydro-lyase (cis-aconitate-forming) activity; mRNA 5ʹ-UTR binding | many biological progresses | apoplast; plasma membrane; plasmodesma; vacuole |
| 5502 (↓) | Aspartate aminotransferase | l-aspartate:2-oxoglutarate aminotransferase activity; l-phenylalanine:2-oxoglutarate aminotransferase activity;  pyridoxal phosphate binding | biosynthetic process; cellular amino acid metabolic process |  |
| 5708 (↑) | Ribulose bisphosphate carboxylase large chain, putative | magnesium ion binding; ribulose-bisphosphate carboxylase activity | carbon fixation |  |
| 5807 (W) | Ribulose bisphosphate carboxylase large chain | GTPase activity; GTP binding; translation elongation factor activity | Protein biosynthesis |  |
| 6004 (↓) | Elongation factor | ATP binding; proton-transporting ATPase activity, rotational mechanism; proton-transporting ATP synthase activity, rotational mechanism | ATP hydrolysis coupled proton transport; plasma membrane ATP synthesis coupled proton transport | chloroplast thylakoid membrane; proton-transporting ATP synthase complex, catalytic core F(1) |
| 6715 (↑) | Ribulose bisphosphate carboxylase large chain, putative | magnesium ion binding; belongs to the RuBisCO large chain family |  |  |
| 6807 (↑) | Os01g0791033 protein | magnesium ion binding; ribulose-bisphosphate carboxylase activity; structural constituent of ribosome | carbon fixation; translation | ribosome |
| 7708 (R) | ATP synthase subunit beta, chloroplastic | ATP binding; proton-transporting ATP synthase activity, rotational mechanism | ATP hydrolysis-coupled proton transport; plasma membrane ATP synthesis-coupled proton transport | chloroplast thylakoid membrane; plastid; proton-transporting ATP synthase complex, catalytic core F(1) |
| 8109 (↓) | Chloroplastic outer envelope membrane protein, putative |  | protein transport | chloroplast outer membrane ; integral component of membrane |
| 8603 (↑) | Ribulose bisphosphate carboxylase large chain, putative | ribulose-bisphosphate carboxylase activity; structural constituent of ribosome | carbon fixation; translation | ribosome |
| 9602 (R) | Phosphoinositide phospholipase | calcium ion binding; phosphatidylinositol phospholipase C activity; signal transducer activity | intracellular signal transduction; lipid catabolic process |  |

Note: (↑) represents up-regulation in rolled leaf mutants (Rolled), (↓) represents down-regulation in rolled leaf mutants, (R) suggests that the protein is only expressed in rolled leaf mutants, (W) suggests that the protein is only expressed in MH86 cultivated rice plants (WT).
