# Supplementary material for: Analysis of the differential gene and protein expression profile of the rolled leaf mutant of transgenic rice (Oryza sativa L.)
Source: PLoS One. 2017 Jul 19;12(7):e0181378. doi: 10.1371/journal.pone.0181378 (PMC5517006; doi:10.1371/journal.pone.0181378)
Supplement: S4 Table — (DOCX) [file pone.0181378.s005.docx]

**S4 Table. Summary of the data obtained from high-throughput sequencing.**

| **Sample name** | **Raw reads** | **Clean reads** | **Clean bases** | **Error rate (%)** | **Q20 (%)** | **Q30 (%)** | **GC content (%)** |
| --- | --- | --- | --- | --- | --- | --- | --- |
| Rolled | 9814084 | 9447511 | 0.94G | 0.06 | 94.05 | 85.31 | 56.51 |
| Unrolled | 10374771 | 10004493 | 1G | 0.06 | 94.36 | 85.90 | 55.75 |
| WT | 13095468 | 12606830 | 1.26G | 0.06 | 94.05 | 85.32 | 56.16 |

**Note**: Raw reads: statistics on raw sequence data; Clean reads: filtered sequencing data. Subsequent bioinformatics analysis is based on clean reads. Clean bases: sequence number is multiplied by the length of sequencing sequence, and the result is converted to consider G as the unit. Error rate: the rate of error. Q20, Q30: the percentage of bases with Phred values greater than 20 and 30 in total bases are calculated, respectively. GC content: the percentage of the total number of bases G and C out of the total number of bases.
