# Supplementary material for: Analysis of the differential gene and protein expression profile of the rolled leaf mutant of transgenic rice (Oryza sativa L.)
Source: PLoS One. 2017 Jul 19;12(7):e0181378. doi: 10.1371/journal.pone.0181378 (PMC5517006; doi:10.1371/journal.pone.0181378)
Supplement: S5 Table — (DOCX) [file pone.0181378.s006.docx]

**S5 Table. Sequence alignment of reads to the reference genome.**

| **Sample name** | **Rolled** | **Unrolled** | **WT** |
| --- | --- | --- | --- |
| Total reads | 9447511 | 10004493 | 12606830 |
| Total mapped | 7949918 (84.15%) | 8674397 (86.71%) | 10817380 (85.81%) |
| Multiple mapped | 317287 (3.36%) | 370824 (3.71%) | 451231 (3.58%) |
| Uniquely mapped | 7632631 (80.79%) | 8303573 (83%) | 10366149 (82.23%) |
| Reads map to ‘+’ | 3809480 (40.32%) | 4143000 (41.41%) | 5170805 (41.02%) |
| Reads map to ‘-’ | 3823151 (40.47%) | 4160573 (41.59%) | 5195344 (41.21%) |
| Non-splice reads | 5736771 (60.72%) | 6132168 (61.29%) | 7803046 (61.9%) |
| Splice reads | 1895860 (20.07%) | 2171405 (21.7%) | 2563103 (20.33%) |

**Note**: Total reads: all clean reads; Total mapped: statistics on reads that can mapped to the reference genome; Multiple mapped: statistics on reads that can mapped to more than one site of the reference genome; Uniquely mapped: statistics on reads that can mapped to a single site of the reference genome; Reads map to ‘+,’ Reads map to ‘-’: statistics on reads that mapped to the plus and minus strands of the reference genome; Splice reads: statistics on reads that mapped to two exons; Non-splice reads are statistics on reads that mapped to the exon with the entire fragment.
