# Supplementary material for: Analysis of the differential gene and protein expression profile of the rolled leaf mutant of transgenic rice (Oryza sativa L.)
Source: PLoS One. 2017 Jul 19;12(7):e0181378. doi: 10.1371/journal.pone.0181378 (PMC5517006; doi:10.1371/journal.pone.0181378)
Supplement: S6 Table — (DOCX) [file pone.0181378.s007.docx]

**S6 Table. Statistical analysis of differentially expressed genes.**

| **Gene_id** | **readcount_**  **Unrolled** | **readcount_**  **Rolled** | **log2.Fold_change** | ***p* value** | **q value** |
| --- | --- | --- | --- | --- | --- |
| BGIOSGA004822 | 12.57622768 | 51.53231683 | -2.0348 | 3.35E-06 | 5.69E-05 |
| BGIOSGA006323 | 17.46698289 | 3.011628906 | 2.536 | 0.00037519 | 0.0040724 |
| BGIOSGA011125 | 122.8278237 | 56.88632378 | 1.1105 | 1.32E-08 | 3.26E-07 |
| BGIOSGA011503 | 131.7709189 | 35.97223415 | 1.8731 | 1.44E-16 | 7.56E-15 |
| BGIOSGA011504 | 298.8950112 | 85.16217295 | 1.8114 | 3.35E-34 | 4.74E-32 |
| BGIOSGA013555 | 129.8146168 | 36.30685959 | 1.8381 | 5.17E-16 | 2.65E-14 |
| BGIOSGA016760 | 242.1622508 | 70.60596657 | 1.7781 | 1.94E-27 | 1.92E-25 |
| BGIOSGA020820 | 28.7855878 | 158.6124557 | -2.4621 | 1.47E-20 | 9.97E-19 |
| BGIOSGA021464 | 16.34909598 | 47.18218619 | -1.529 | 0.00038901 | 0.004198 |
| BGIOSGA022210 | 100.3303497 | 25.26422027 | 1.9896 | 8.03E-14 | 3.46E-12 |
| BGIOSGA023953 | 27.24849331 | 72.44640646 | -1.4107 | 4.13E-05 | 0.00056251 |
| BGIOSGA024059 | 56.73276043 | 131.842421 | -1.2166 | 1.33E-06 | 2.43E-05 |
| BGIOSGA028855 | 60.08642114 | 158.6124557 | -1.4004 | 1.66E-09 | 4.58E-08 |
| BGIOSGA028962 | 220.0839844 | 77.63310069 | 1.5033 | 1.28E-20 | 8.77E-19 |

**Note:** Gene_id: gene number; readcount_Sample1: calibrated readcount value of Sample1; readcount_Sample2: calibrated readcount value of Sample2; log2FoldChange: log2 (Sample1/Sample2); p value (pval): statistically significant test indicators; q value (padj): calibrated *p* value. The smaller is the q value, the more significant is the difference of gene expression.
